# Supplementary material for: Enzyme activities predicted by metabolite concentrations and solvent capacity in the cell
Source: J R Soc Interface. 2020 Oct 14;17(171):20200656. doi: 10.1098/rsif.2020.0656 (PMC7653389; doi:10.1098/rsif.2020.0656)
Supplement: Supplementary Material [file rsif20200656supp1.pdf]

Supplementary Materials

Models and Source code

All models and code implementing the procedures described in the main text and below is available as either computational notebooks (Jupyter notebooks) or python code at https://github.com/sambritton/Python_Notebook_Regulation.

Model training

Reinforcement learning agents are trained by iteratively learning the value function of each state, s_t. The value at a state represents the expected reward to be achieved from following the current policy: $V(s_t) = E[r_{t:t+n}|s_t]$. At each state with $t \geq n$ the squared error between the value of the state, $V(s_t)$, and the experienced rewards, $r_{t:t+n}$, is back-propagated to calculate appropriate changes in the neural network weights. As agents explore different possible regulation schemes, rewards are accumulated and averaged over each episode of training. Average rewards per episode are shown over the 350 training episodes for the gluconeogenesis and glycolysis-TCA pathways (Figure S1A) as well as the glycolysis-PPP-TCA pathway for each of the environmental conditions (Figure S1B).

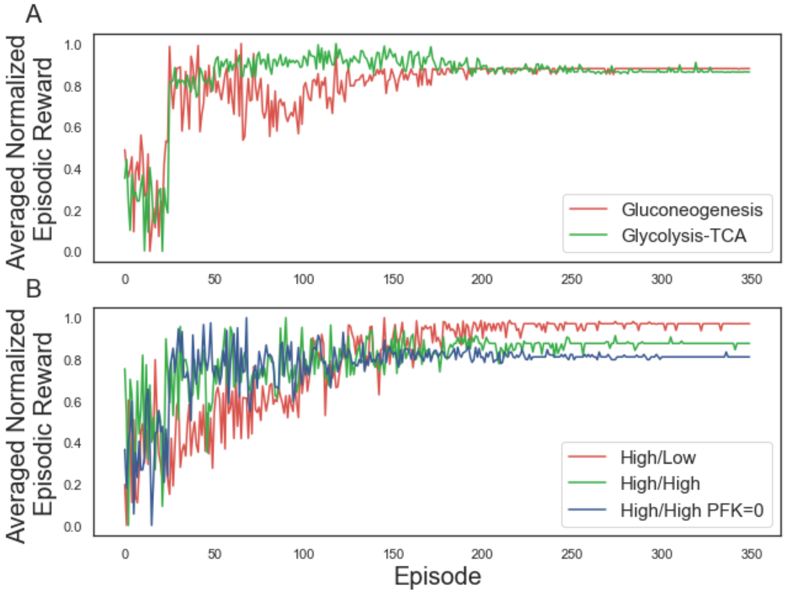

Figure S1. Cumulative normalized rewards averaged over 10 RL runs for the hyper-parameters (n, lr) which resulted in the maximal reward.

Calculating concentration control coefficients

Enzyme activities begin from a value of 1.0, i.e. the enzyme is unregulated. The current value of the activity is adjusted using Metabolic Control Analysis (MCA)[1]. In MCA, the concentration control coefficient is found by first computing the M by M symmetric linear stability matrix, A^{n^*}, given by[1],

$$A_{ij}^{n^*} = n_j^* \sum_{k=1}^{Z} S_{ik} \frac{\partial J_k}{\partial n_j}|_{n=n*}. \tag{1}$$

Here n_i^* is the current concentration of metabolite i, S is the stoichiometric matrix and J is the vector of reaction fluxes. The concentration control coefficient for metabolite i due to reaction j is then,

$$\begin{aligned} C_{i,j}^{n} &= \frac{\partial \log n_i}{\partial \log \alpha_j} \\ &= \frac{\alpha_j}{n_i^*} \frac{\Delta n_i}{\Delta \alpha_j} \\ &= -(BS)_{ij} J_j, \end{aligned} \tag{2}$$

where $B = A^{-1}$. Note that the calculation of $C^n_{i,j}$ assumes metabolite concentrations are linearly dependent on enzyme activities. This assumption can be used to isolate the change in activity, j, needed to make a change in the product concentration n_i:

$$\Delta\alpha_j = \alpha_j \frac{\Delta n_i}{n_i^*}\left(-\sum_{k=1}^{Z} B_{ik} S_{kj} J_j\right). \tag{3}$$

In practice, when $C^n_{i,j} >> 0.0$ the assumption of a small change n_i used in MCA is no longer valid and instead the current activity j is instead updated using $\alpha_{j,new} = \alpha_{j,current}/5$. As the cost function L (Methods Eqn. 20) approaches zero, then Eqn. 3 can be applied.

Analysis of gluconeogenesis pathway

The gluconeogenesis pathway is analyzed at low NAD/NADH ratio (0.02). The pathway has two known regulation sites fructose 1,6-bisphosphatase (FBP) and pyruvate carboxylase (PC). While both can be utilized to bring steady state metabolite concentrations into agreement with experimentally observed values, regulation of pyruvate carboxylase results in a larger energy dissipation rate (dE/dt). Regulation of alternative enzymes results in lower flux through the pathway and less energy available for use. Optimal predicted enzyme activities are in agreement for each method (Figure S2A). Table S4 lists the complete reaction activity, flux and free energy for each respective prediction method.

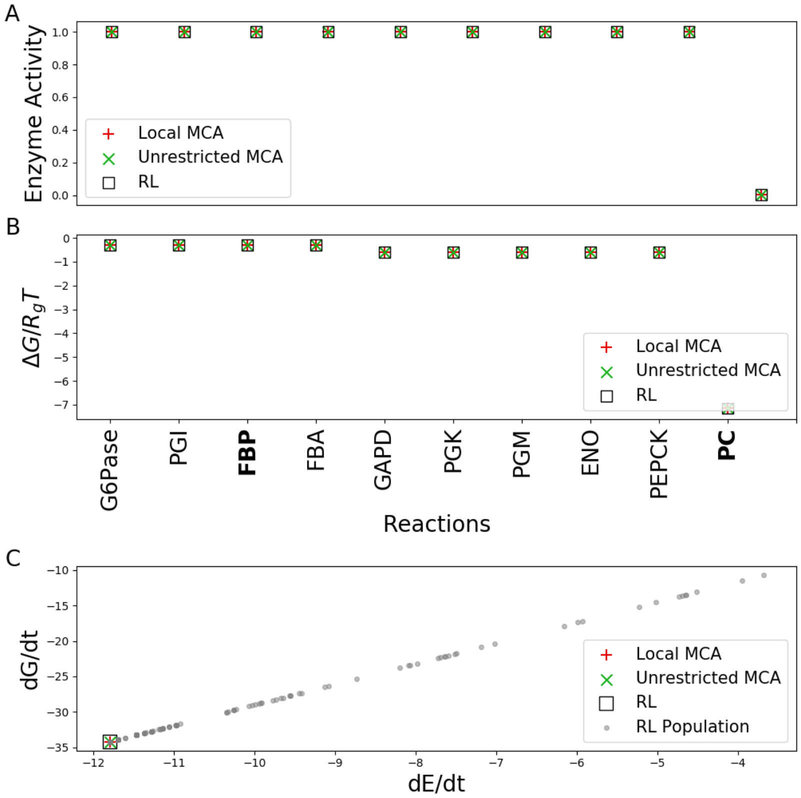

Figure S2. Gluconeogenesis cycle predictions with low NAD/NADH initial conditions. Predicted enzyme activities (A) and free energy (B) at terminal states are calculated using concentration control theory, shown as red 'plus's and green 'X's, respectively. Results are compared to those found using a RL approach (black square). Grey dots (C) represent the population of terminal states found while training the RL agent.

Analysis of glycolysis-TCA pathway

The glycolysis-TCA pathway is a subset of the larger glycolysis-PPP-TCA pathway discussed in the Results which includes the pentose phosphate pathway (PPP). Reducing the number of reactions limits the possible regulation schemes. When utilizing the same initial metabolite concentrations as the larger pathway when appropriate, i.e. high NAD/NADH (31.3), the regulation schemes for various methods show closer agreement. Both HEX1 and GAPD are regulated by every method as in the glycolysis-PPP-TCA pathway. The local MCA method, however, regulates PFK, PGK, and PDH, while the RL method

additionally regulates PGI. Both methods regulate more reactions than the unrestriced MCA method and therefore result in a lower energy dissipation rate. Table S5 lists the complete reaction activity, flux and free energy for each respective prediction method.

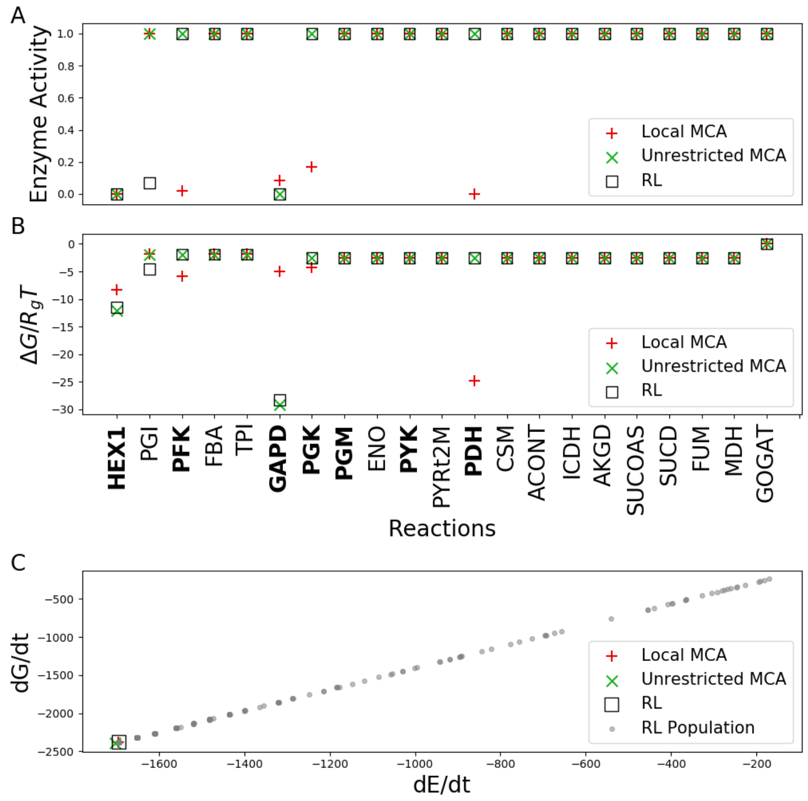

Figure S3. Glycolysis-TCA cycle predictions with high NAD/NADH initial conditions. Predicted enzyme activities (A) and free energy (B) at terminal states are calculated using concentration control theory, shown as red ‘plus’s and green ‘X’s, respectively. Results are compared to those found using a RL approach (black square). Grey dots (C) represent the population of terminal states found while training the RL agent.

Analysis of pathways with proxy data

When no metabolomics data is available, the methods presented here are still able to perform accurate predictive measurements in terms of enzyme regulation, steady-state metabolite concentrations and reaction flux. Instead of utilizing known metabolomics data measurements, we instead assume the target values of previously measured variable metabolites are fixed at 0.1 mM. Predictive learning is performed for the glycolysis-TCA cycle (Figure S4) and glycolysis-PPP-TCA pathway under the same three initial conditions (Figures S5, S6 and S7). In all initial conditions, for both pathways, the unrestricted MCA method maintains the same regulation of enzymes GAPD and HEX1. Variations occur only in the amount of regulation applied to the respective reactions. The other two methods show more variation. In the glycolysis-TCA cycle, the local MCA method regulates PYK in addition to the reactions previously regulated, while the RL method predicts additional regulation to PFK and PGM (Figure S4).

The glycolysis-PPP-TCA pathway, on the other hand, shows more variation. Specifically, in the high NAD/NADH and low NADP/NADPH ratio initial condition, the local MCA regulates PYK in addition to the reactions previously regulated. The RL method predicts increased regulation to PGI and PGM but no longer regulates PDH (Figure S5). Under the high NAD/NADH and high NADP/NADPH ratio initial conditions, both the local MCA and RL methods predict regulation schemes with additional reduction in activity of PYK. The RL additionally regulates G6PDH, while neither method regulates TKT1 or PYRt2m (Figure S6). Only slight alterations are observed in enzyme activity when PFK has zero activity (Figure S7).

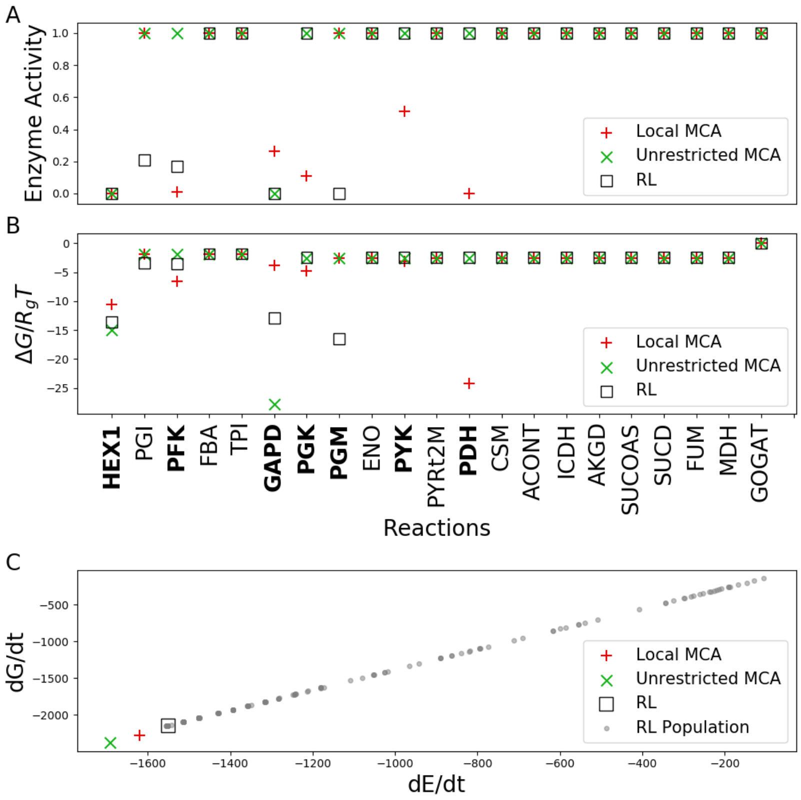

Figure S4. Glycolysis-TCA cycle predictions with high NAD/NADH initial conditions without experimental metabolomics data. Predicted enzyme activities (A) and free energy (B) at terminal states are calculated using concentration control theory, shown as red 'plus's and green 'X's, respectively. Results are compared to those found using a RL approach (black square). Grey dots (C) represent the population of terminal states found while training the RL agent.

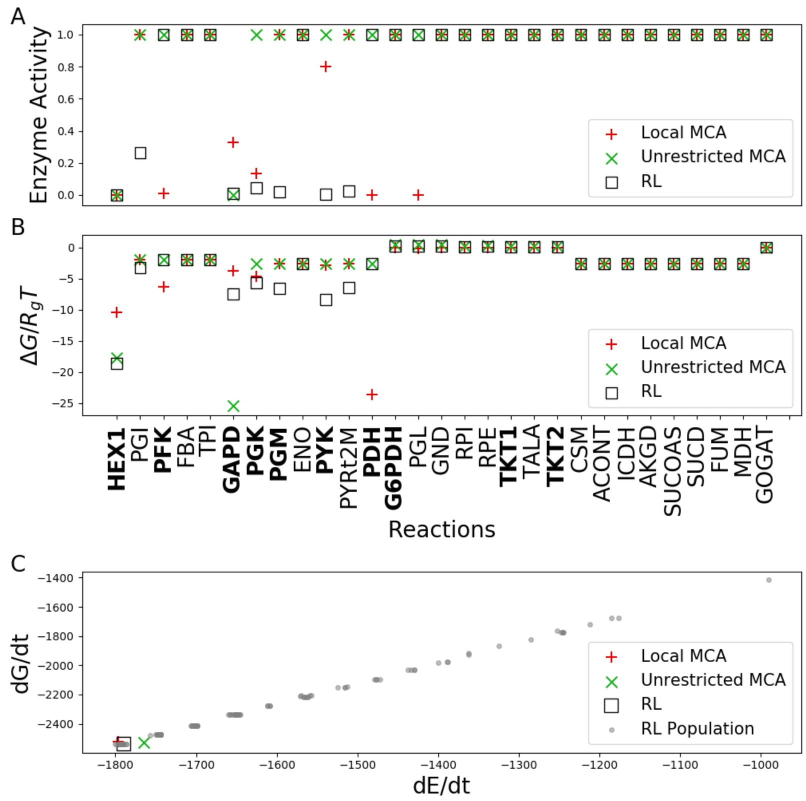

Figure S5. Glycolysis-PPP-TCA cycle predictions with high NAD/NADH and low NADP/NADPH conditions without experimental metabolomics data. (A) Predicted enzyme activities at terminal states are calculated using Metabolic Control Analysis, shown as red 'plus's and green 'X's, respectively. Results are compared to those found using a RL approach (black square). (B) Reaction free energies. (C) Free energy and energy dissipation rates. Grey dots represent the population of terminal states found while training the RL agent.

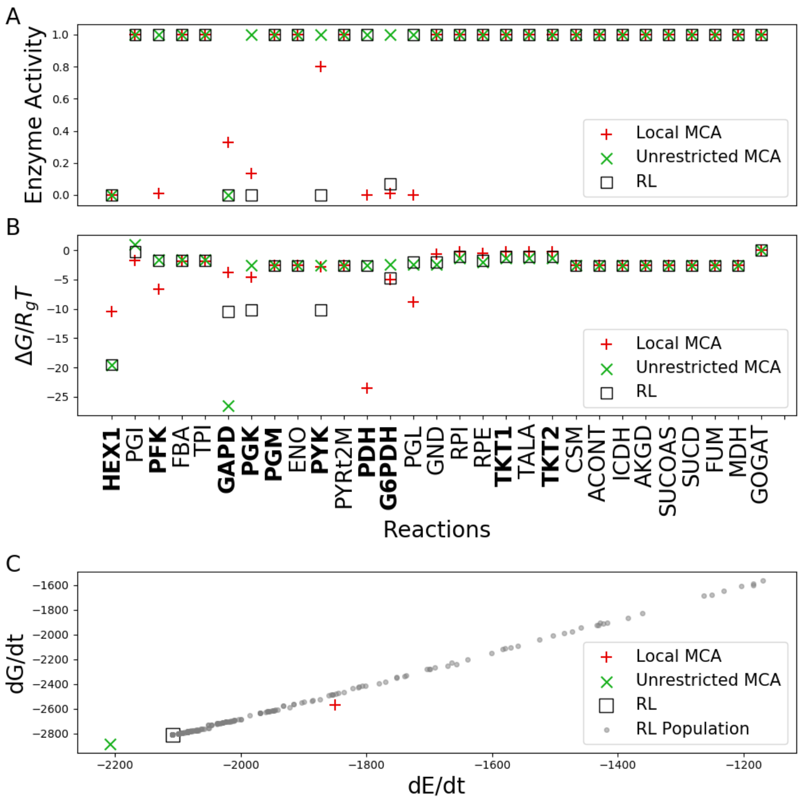

Figure S6. Glycolysis-PPP-TCA cycle predictions with high NAD/NADH and high NADP/NADPH conditions without experimental metabolomics data. (A) Predicted enzyme activities at terminal states are calculated using Metabolic Control Analysis, shown as red ‘plus’s and green ‘X’s, respectively. Results are compared to those found using a RL approach (black square). (B) Reaction free energies. (C) Free energy and energy dissipation rates. Grey dots represent the population of terminal states found while training the RL agent.

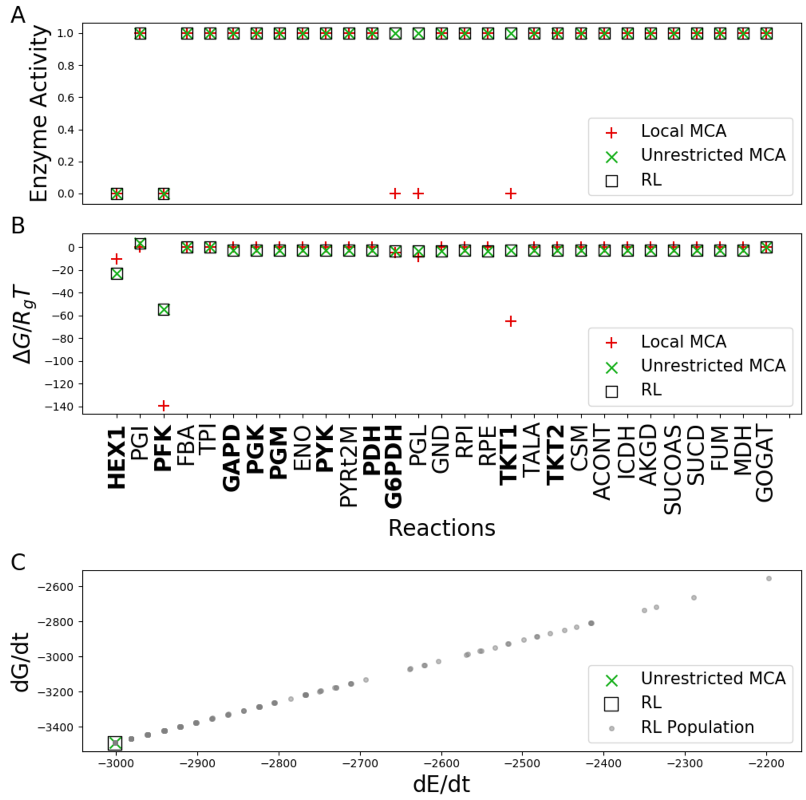

Figure S7. Glycolysis-PPP-TCA cycle predictions with high NAD/NADH and high NADP/NADPH conditions and PFK activity set to zero without experimental metabolomics data. (A) Predicted enzyme activities at terminal states are calculated using Metabolic Control Analysis, shown as red 'plus's and green 'X's, respectively. Results are compared to those found using a RL approach (black square). (B) Reaction free energies. (C) Free energy and energy dissipation rates. Grey dots represent the population of terminal states found while training the RL agent. The local MCA method results in zero flux as in the case when experimental data is utilized and is therefore not shown.

Tables

NAD/NADH // NADP/NADPH Ratio		High//Low			High//High			High//High no PFK		
Method		MCA Local	MCA	RL	MCA Local	MCA	RL	MCA Local	MCA	RL
Glycolysis	HEX1	6.34E+00	6.25E+00	6.28E+00	6.27E+00	8.23E+00	8.22E+00	5.76E-03	1.22E+01	1.22E+01
	PGI	6.34E+00	7.05E+00	7.21E+00	6.19E+00	-2.80E+0	-2.40E+00	-1.15E-02	-2.44E+01	-2.44E+01
	PFK	6.34E+00	6.52E+00	6.59E+00	6.24E+00	4.56E+00	4.68E+00	0.00E+00	0.00E+00	0.00E+00
	FBA	6.34E+00	6.52E+00	6.59E+00	6.24E+00	4.56E+00	4.68E+00	1.05E-14	-3.80E-15	-3.80E-15
	TPI	6.34E+00	6.52E+00	6.59E+00	6.24E+00	4.56E+00	4.68E+00	-7.80E-16	6.22E-15	-7.80E-16
	GAPD	1.27E+01	1.28E+01	1.29E+01	1.25E+01	1.28E+01	1.29E+01	5.76E-03	1.22E+01	1.22E+01
	PGK	1.27E+01	1.28E+01	1.29E+01	1.25E+01	1.28E+01	1.29E+01	5.76E-03	1.22E+01	1.22E+01
	PGM	1.27E+01	1.28E+01	1.29E+01	1.25E+01	1.28E+01	1.29E+01	5.76E-03	1.22E+01	1.22E+01
	ENO	1.27E+01	1.28E+01	1.29E+01	1.25E+01	1.28E+01	1.29E+01	5.76E-03	1.22E+01	1.22E+01
	PYK	1.27E+01	1.28E+01	1.29E+01	1.25E+01	1.28E+01	1.29E+01	5.76E-03	1.22E+01	1.22E+01
	PYRt2m	1.27E+01	1.28E+01	1.29E+01	1.25E+01	1.28E+01	1.29E+01	5.76E-03	1.22E+01	1.22E+01
	PDH	1.27E+01	1.28E+01	1.29E+01	1.25E+01	1.28E+01	1.29E+01	5.76E-03	1.22E+01	1.22E+01
PPP	G6PDH	2.56E-14	-7.98E-01	-9.30E-01	8.09E-02	1.10E+01	1.06E+01	1.73E-02	3.66E+01	3.66E+01
	PGL	5.27E-33	-7.98E-01	-9.30E-01	8.09E-02	1.10E+01	1.06E+01	1.73E-02	3.66E+01	3.66E+01
	GND	3.18E-14	-7.98E-01	-9.30E-01	8.09E-02	1.10E+01	1.06E+01	1.73E-02	3.66E+01	3.66E+01
	RPI	8.10E-15	-2.66E-01	-3.10E-01	2.70E-02	3.68E+00	3.54E+00	5.76E-03	1.22E+01	1.22E+01
	RPE	1.42E-14	-5.32E-01	-6.20E-01	5.39E-02	7.36E+00	7.08E+00	1.15E-02	2.44E+01	2.44E+01
	TKT1	4.88E-15	-2.66E-01	-3.10E-01	2.70E-02	3.68E+00	3.54E+00	5.76E-03	1.22E+01	1.22E+01
	TALA	9.77E-15	-2.66E-01	-3.10E-01	2.70E-02	3.68E+00	3.54E+00	5.76E-03	1.22E+01	1.22E+01
	TKT2	1.07E-14	-2.66E-01	-3.10E-01	2.70E-02	3.68E+00	3.54E+00	5.76E-03	1.22E+01	1.22E+01
TCA	CSM	1.27E+01	1.28E+01	1.29E+01	1.25E+01	1.28E+01	1.29E+01	5.76E-03	1.22E+01	1.22E+01
	ACONT	1.27E+01	1.28E+01	1.29E+01	1.25E+01	1.28E+01	1.29E+01	5.76E-03	1.22E+01	1.22E+01
	ICDH	1.27E+01	1.28E+01	1.29E+01	1.25E+01	1.28E+01	1.29E+01	5.76E-03	1.22E+01	1.22E+01
	AKGD	1.27E+01	1.28E+01	1.29E+01	1.25E+01	1.28E+01	1.29E+01	5.76E-03	1.22E+01	1.22E+01
	SUCOAS	1.27E+01	1.28E+01	1.29E+01	1.25E+01	1.28E+01	1.29E+01	5.76E-03	1.22E+01	1.22E+01
	SUCD	1.27E+01	1.28E+01	1.29E+01	1.25E+01	1.28E+01	1.29E+01	5.76E-03	1.22E+01	1.22E+01
	FUM	1.27E+01	1.28E+01	1.29E+01	1.25E+01	1.28E+01	1.29E+01	5.76E-03	1.22E+01	1.22E+01
	MDH	1.27E+01	1.28E+01	1.29E+01	1.25E+01	1.28E+01	1.29E+01	5.76E-03	1.22E+01	1.22E+01
	GOGAT	6.99E-15	6.99E-15	-1.10E-16	-1.10E-16	-1.10E-16	-1.10E-16	-1.10E-16	-1.10E-16	-1.10E-16

Table S1. Reaction fluxes at predicted enzyme activities from MCA-local, MCA, and RL methods for the glycolysis-PPP-TCA pathway under different boundary conditions. Under unregulated conditions (main text, Figure 2A) and under High/Low conditions, flux through lower glycolysis and the TCA cycle is twice that of upper glycolysis. The reason for this is that for each molecule of glucose entering upper glycolysis, two molecules of glyceraldehyde 3-phosphate enter lower glycolysis (and consequently, the TCA cycle). Comparing across boundary conditions, the flux through HEX1, the entry point for carbon, is slightly higher under High/High conditions compared to the High/Low condition. This increased flux, however, does not flow into upper glycolysis but rather into the PPP (HEX1 is necessary for both upper glycolysis and PPP), a pathway that was not thermodynamically feasible under High/Low conditions. That is, flux at HEX1 appears to be ‘up-regulated’ in High/High conditions relative to High/Low conditions, but the reason for this is the more favorable overall thermodynamics of the High/High condition and cannot be attributed directly to regulation.

NAD/NADH // NADP/NADPH Ratio		High//Low			High//High			High//High no PFK		
Method		MCA Local	MCA	RL	MCA Local	MCA	RL	MCA Local	MCA	RL
Glycolysis	HEX1	-8.32E+00	-1.63E+01	-1.79E+01	-8.31E+00	-1.82E+01	-2.00E+01	-8.23E+00	-2.28E+01	-2.28E+01
	PGI	-1.87E+00	-1.97E+00	-1.99E+00	-1.85E+00	1.14E+00	1.01E+00	5.76E-03	3.19E+00	3.19E+00
	PFK	-5.86E+00	-1.90E+00	-1.91E+00	-5.85E+00	-1.56E+00	-1.59E+00	-1.41E+02	-5.49E+01	-5.49E+01
	FBA	-1.87E+00	-1.90E+00	-1.91E+00	-1.86E+00	-1.56E+00	-1.59E+00	-5.30E-15	1.78E-15	1.78E-15
	TPI	-1.87E+00	-1.90E+00	-1.91E+00	-1.86E+00	-1.56E+00	-1.59E+00	3.33E-16	-3.10E-15	3.33E-16
	GAPD	-4.99E+00	-2.71E+01	-1.04E+01	-5.20E+00	-2.82E+01	-1.33E+01	-2.88E-03	-2.51E+00	-2.51E+00
	PGK	-4.32E+00	-2.55E+00	-9.92E+00	-4.31E+00	-2.55E+00	-1.28E+01	-2.88E-03	-2.51E+00	-2.51E+00
	PGM	-2.55E+00	-2.55E+00	-2.56E+00	-2.53E+00	-2.55E+00	-2.56E+00	-2.88E-03	-2.51E+00	-2.51E+00
	ENO	-2.55E+00	-2.55E+00	-2.56E+00	-2.53E+00	-2.55E+00	-2.56E+00	-2.88E-03	-2.51E+00	-2.51E+00
	PYK	-2.55E+00	-2.55E+00	-5.90E+00	-2.53E+00	-2.55E+00	-2.56E+00	-2.88E-03	-2.51E+00	-2.51E+00
	PYRt2m	-2.55E+00	-2.55E+00	-7.02E+00	-2.53E+00	-2.55E+00	-6.13E+00	-2.88E-03	-2.51E+00	-2.51E+00
	PDH	-2.51E+01	-2.55E+00	-3.23E+00	-2.51E+01	-2.55E+00	-2.56E+00	-2.88E-03	-2.51E+00	-2.51E+00
PPP	G6PDH	-1.30E-14	3.89E-01	4.50E-01	-7.08E+00	-2.41E+00	-2.37E+00	-7.10E+00	-3.60E+00	-3.60E+00
	PGL	-3.84E-01	3.89E-01	4.50E-01	-7.53E+00	-2.41E+00	-2.37E+00	-4.20E+00	-3.60E+00	-3.60E+00
	GND	-1.60E-14	3.89E-01	4.50E-01	-4.04E-02	-2.41E+00	-2.37E+00	-8.64E-03	-3.60E+00	-3.60E+00
	RPI	-4.00E-15	1.33E-01	1.54E-01	-1.35E-02	-1.37E+00	-1.34E+00	-2.88E-03	-2.51E+00	-2.51E+00
	RPE	-7.10E-15	2.63E-01	3.05E-01	-2.70E-02	-2.01E+00	-1.98E+00	-5.76E-03	-3.19E+00	-3.19E+00
	TKT1	-2.40E-15	1.33E-01	1.54E-01	-1.98E+00	-1.37E+00	-1.34E+00	-7.34E+01	-2.51E+00	-2.51E+00
	TALA	-4.90E-15	1.33E-01	1.54E-01	-1.35E-02	-1.37E+00	-1.34E+00	-2.88E-03	-2.51E+00	-2.51E+00
	TKT2	-5.30E-15	1.33E-01	1.54E-01	-1.35E-02	-1.37E+00	-1.34E+00	-2.88E-03	-2.51E+00	-2.51E+00
TCA	CSM	-2.55E+00	-2.55E+00	-2.56E+00	-2.53E+00	-2.55E+00	-2.56E+00	-2.88E-03	-2.51E+00	-2.51E+00
	ACONT	-2.55E+00	-2.55E+00	-2.56E+00	-2.53E+00	-2.55E+00	-2.56E+00	-2.88E-03	-2.51E+00	-2.51E+00
	ICDH	-2.55E+00	-2.55E+00	-2.56E+00	-2.53E+00	-2.55E+00	-2.56E+00	-2.88E-03	-2.51E+00	-2.51E+00
	AKGD	-2.55E+00	-2.55E+00	-2.56E+00	-2.53E+00	-2.55E+00	-2.56E+00	-2.88E-03	-2.51E+00	-2.51E+00
	SUCOAS	-2.55E+00	-2.55E+00	-2.56E+00	-2.53E+00	-2.55E+00	-2.56E+00	-2.88E-03	-2.51E+00	-2.51E+00
	SUCD	-2.55E+00	-2.55E+00	-2.56E+00	-2.53E+00	-2.55E+00	-2.56E+00	-2.88E-03	-2.51E+00	-2.51E+00
	FUM	-2.55E+00	-2.55E+00	-2.56E+00	-2.53E+00	-2.55E+00	-2.56E+00	-2.88E-03	-2.51E+00	-2.51E+00
	MDH	-2.55E+00	-2.55E+00	-2.56E+00	-2.53E+00	-2.55E+00	-2.56E+00	-2.88E-03	-2.51E+00	-2.51E+00
	GOGAT	-3.60E-15	-3.60E-15	1.11E-16	1.11E-16	1.11E-16	1.11E-16	1.11E-16	1.11E-16	1.11E-16

Table S2. Reaction free energy at predicted enzyme activities from MCA-local, MCA, and RL methods for the glycolysis-PPP-TCA pathway under different boundary conditions.

NAD/NADH // NADP/NADPH Ratio		High//Low			High//High			High//High no PFK		
Method		MCA Local	MCA	RL	MCA Local	MCA	RL	MCA Local	MCA	RL
Glycolysis	HEX1	1.55E-03	5.02E-07	1.05E-07	1.55E-03	1.55E-03	1.77E-08	1.23E-06	1.52E-09	1.52E-09
	PFK	1.80E-02	1.00E+00	1.00E+00	1.80E-02	1.00E+00	1.00E+00	0.00E+00	0.00E+00	0.00E+00
	GAPD	8.59E-02	2.19E-11	4.06E-04	6.87E-02	7.17E-12	2.23E-05	1.00E+00	1.00E+00	1.00E+00
	PGK	1.68E-01	1.00E+00	6.34E-04	1.68E-01	1.00E+00	3.48E-05	1.00E+00	1.00E+00	1.00E+00
	PYK	1.00E+00	1.00E+00	3.52E-02	1.00E+00	1.00E+00	1.00E+00	1.00E+00	1.00E+00	1.00E+00
	PYRt2m	1.00E+00	1.00E+00	1.15E-02	1.00E+00	1.00E+00	2.81E-02	1.00E+00	1.00E+00	1.00E+00
	PDH	1.63E-10	1.00E+00	5.12E-01	1.63E-10	1.00E+00	1.00E+00	1.00E+00	1.00E+00	1.00E+00
PPP	G6PDH	1.00E+00	1.00E+00	1.00E+00	6.81E-05	1.00E+00	1.00E+00	1.43E-05	1.00E+00	1.00E+00
	PGL	5.36E-33	1.00E+00	1.00E+00	4.36E-05	1.00E+00	1.00E+00	2.60E-04	1.00E+00	1.00E+00
	TKT1	1.00E+00	1.00E+00	1.00E+00	3.78E-03	1.00E+00	1.00E+00	7.72E-35	1.00E+00	1.00E+00

Table S3. Predicted enzyme activities from MCA-local, MCA, and RL methods for the glycolysis-PPP-TCA pathway under different boundary conditions. Unlisted reactions are unregulated.

		Activity			Flux			Energy		
Method		MCA Local	MCA	RL	MCA Local	MCA	RL	MCA Local	MCA	RL
Gluconeogenesis	G6Pase	1.00E+00	1.00E+00	1.00E+00	6.33E-01	6.33E-01	6.33E-01	-3.11E-01	-3.11E-01	-3.11E-01
	PGI	1.00E+00	1.00E+00	1.00E+00	6.33E-01	6.33E-01	6.33E-01	-3.11E-01	-3.11E-01	-3.11E-01
	FBP	1.00E+00	1.00E+00	1.00E+00	6.33E-01	6.33E-01	6.33E-01	-3.11E-01	-3.11E-01	-3.11E-01
	FBA	1.00E+00	1.00E+00	1.00E+00	6.33E-01	6.33E-01	6.33E-01	-3.11E-01	-3.11E-01	-3.11E-01
	GAPD	1.00E+00	1.00E+00	1.00E+00	1.27E+00	1.27E+00	1.27E+00	-5.97E-01	-5.97E-01	-5.97E-01
	PGK	1.00E+00	1.00E+00	1.00E+00	1.27E+00	1.27E+00	1.27E+00	-5.97E-01	-5.97E-01	-5.97E-01
	PGM	1.00E+00	1.00E+00	1.00E+00	1.27E+00	1.27E+00	1.27E+00	-5.97E-01	-5.97E-01	-5.97E-01
	ENO	1.00E+00	1.00E+00	1.00E+00	1.27E+00	1.27E+00	1.27E+00	-5.97E-01	-5.97E-01	-5.97E-01
	PEPCK	1.00E+00	1.00E+00	1.00E+00	1.27E+00	1.27E+00	1.27E+00	-5.97E-01	-5.97E-01	-5.97E-01
	PC	9.90E-04	9.90E-04	9.90E-04	1.27E+00	1.27E+00	1.26E+00	-7.15E+00	-7.15E+00	-7.15E+00

Table S4. Predicted enzyme activities, fluxes, and free energy for gluconeogenesis pathway from MCA-local, MCA, and RL methods.

		Activity			Flux			Energy		
Method		MCA Local	MCA	RL	MCA Local	MCA	RL	MCA Local	MCA	RL
Glycolysis	HEX1	1.55E-03	3.48E-05	6.81E-05	6.42E+00	6.45E+00	6.41E+00	-8.33E+00	-1.21E+01	-1.15E+01
	PGI	1.00E+00	1.00E+00	6.87E-02	6.42E+00	6.45E+00	6.41E+00	-1.88E+00	-1.89E+00	-4.54E+00
	PFK	1.80E-02	1.00E+00	1.00E+00	6.42E+00	6.45E+00	6.41E+00	-5.88E+00	-1.89E+00	-1.88E+00
	FBA	1.00E+00	1.00E+00	1.00E+00	6.42E+00	6.45E+00	6.41E+00	-1.88E+00	-1.89E+00	-1.88E+00
	TPI	1.00E+00	1.00E+00	1.00E+00	6.42E+00	6.45E+00	6.41E+00	-1.88E+00	-1.89E+00	-1.88E+00
	GAPD	8.59E-02	2.94E-12	7.17E-12	1.28E+01	1.29E+01	1.28E+01	-5.01E+00	-2.91E+01	-2.82E+01
	PGK	1.68E-01	1.00E+00	1.00E+00	1.28E+01	1.29E+01	1.28E+01	-4.34E+00	-2.56E+00	-2.56E+00
	PGM	1.00E+00	1.00E+00	1.00E+00	1.28E+01	1.29E+01	1.28E+01	-2.56E+00	-2.56E+00	-2.56E+00
	ENO	1.00E+00	1.00E+00	1.00E+00	1.28E+01	1.29E+01	1.28E+01	-2.56E+00	-2.56E+00	-2.56E+00
	PYK	1.00E+00	1.00E+00	1.00E+00	1.28E+01	1.29E+01	1.28E+01	-2.56E+00	-2.56E+00	-2.56E+00
	PYRt2m	1.00E+00	1.00E+00	1.00E+00	1.28E+01	1.29E+01	1.28E+01	-2.56E+00	-2.56E+00	-2.56E+00
	PDH	2.04E-10	1.00E+00	1.00E+00	1.28E+01	1.29E+01	1.28E+01	-2.49E+01	-2.56E+00	-2.56E+00
TCA	CSM	1.00E+00	1.00E+00	1.00E+00	1.28E+01	1.29E+01	1.28E+01	-2.56E+00	-2.56E+00	-2.56E+00
	ACONT	1.00E+00	1.00E+00	1.00E+00	1.28E+01	1.29E+01	1.28E+01	-2.56E+00	-2.56E+00	-2.56E+00
	ICDH	1.00E+00	1.00E+00	1.00E+00	1.28E+01	1.29E+01	1.28E+01	-2.56E+00	-2.56E+00	-2.56E+00
	AKGD	1.00E+00	1.00E+00	1.00E+00	1.28E+01	1.29E+01	1.28E+01	-2.56E+00	-2.56E+00	-2.56E+00
	SUCOAS	1.00E+00	1.00E+00	1.00E+00	1.28E+01	1.29E+01	1.28E+01	-2.56E+00	-2.56E+00	-2.56E+00
	SUCD	1.00E+00	1.00E+00	1.00E+00	1.28E+01	1.29E+01	1.28E+01	-2.56E+00	-2.56E+00	-2.56E+00
	FUM	1.00E+00	1.00E+00	1.00E+00	1.28E+01	1.29E+01	1.28E+01	-2.56E+00	-2.56E+00	-2.56E+00
	MDH	1.00E+00	1.00E+00	1.00E+00	1.28E+01	1.29E+01	1.28E+01	-2.56E+00	-2.56E+00	-2.56E+00
	GOGAT	1.00E+00	1.00E+00	1.00E+00	-1.10E-16	-1.10E-16	-1.10E-16	1.11E-16	1.11E-16	1.11E-16

Table S5. Predicted enzyme activities, fluxes, and free energy for glycolysis-TCA pathway from MCA-local, MCA, and RL methods.

References

1. Beard, D. A. & Qian, H. *Chemical biophysics : quantitative analysis of cellular systems*. Cambridge texts in biomedical engineering (Cambridge University Press, Cambridge ; New York, 2008).
